# Metabolic Aspects of Neuron-Oligodendrocyte-Astrocyte Interactions

**DOI:** 10.3389/fendo.2013.00054

**Published:** 2013-05-13

**Authors:** Ana I. Amaral, Tore W. Meisingset, Mark R. Kotter, Ursula Sonnewald

**Affiliations:** ^1^Anne McLaren Laboratory for Regenerative Medicine, Wellcome Trust and Medical Research Council Cambridge Stem Cell Institute, Department of Clinical Neurosciences, University of CambridgeCambridge, UK; ^2^Department of Neuroscience, Faculty of Medicine, Norwegian University of Science and TechnologyTrondheim, Norway

**Keywords:** energy metabolism, glucose, gray matter, white matter, pyruvate carboxylation, glycolysis, lactate, monocarboxylate transporters

## Abstract

Whereas astrocytes have been in the limelight of scientific interest in brain energy metabolism for a while, oligodendrocytes are still waiting for a place on the metabolic stage. We propose to term the interaction of oligodendrocytes with astrocytes and neurons: NOA (neuron–oligodendrocyte–astrocyte) interactions. One of the reasons to find out more about metabolic interactions between oligodendrocytes, neurons, and astrocytes is to establish markers of healthy oligodendrocyte metabolism that could be used for the diagnosis and assessment of white matter disease. The vesicular release of glutamate in the white matter has received considerable attention in the past. Oligodendrocyte lineage cells express glutamate receptors and glutamate toxicity has been implicated in diseases affecting oligodendrocytes such as hypoxic-ischaemic encephalopathy, inflammatory diseases and trauma. As oligodendrocyte precursor cells vividly react to injury it is also important to establish whether cells recruited into damaged areas are able to regenerate lost myelin sheaths or whether astrocytic scarring occurs. It is therefore important to consider metabolic aspects of astrocytes and oligodendrocytes separately. The present review summarizes the limited evidence available on metabolic cycles in oligodendrocytes and so hopes to stimulate further research interests in this important field.

## Introduction

The body of research from the last century has established the present view of contiguous neurons in continuous, dynamic interaction with several types of glial cells (astrocytes, oligodendrocytes, microglia). The interactions between neurons and astrocytes characterized by the glutamate–glutamine(–GABA) shuttle have received considerable attention since its discovery in the 1970s (van den Berg and Garfinkel, 1971). This shuttle is necessary since neurons cannot make their amino acid neurotransmitters glutamate (excitatory, 90% of synapses), GABA (most abundant inhibitory), and aspartate without glutamine from astrocytes. In this interplay, glucose has a central role as the major (or exclusive) source of energy for the adult brain and the molecules used to synthesize glutamine and thus glutamate, GABA, and aspartate (McKenna et al., 2011).

**Figure 1:**
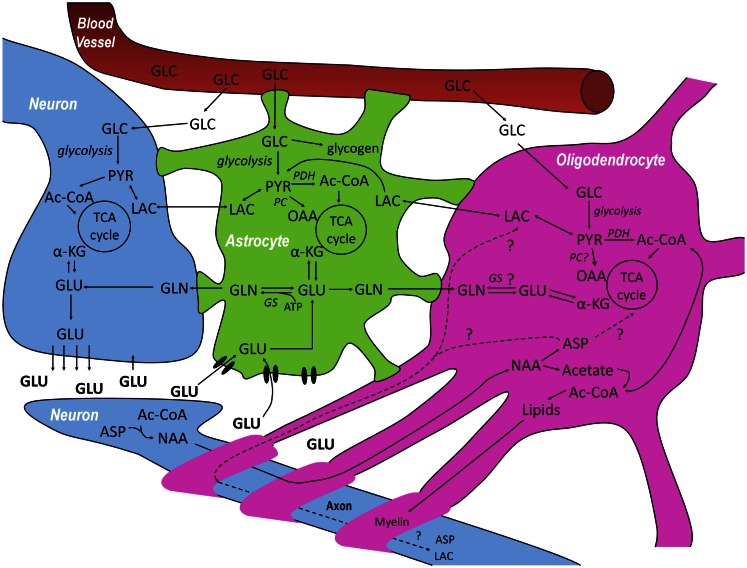
**Schematic overview of the metabolic interactions between neurons–oligodendrocytes–astrocytes (NOA) discussed in this review**. Glucose (GLC) from the blood is taken up by neurons, astrocytes, and oligodendrocytes and can be metabolized via glycolysis, giving rise to pyruvate (PYR). In astrocytes, GLC can also be stored in the form of glycogen. PYR, the end product of glycolysis can be reduced to lactate (LAC) which can be released and taken up by cells with lower lactate concentration, be converted into alanine (not shown) or be converted into acetyl-CoA (Ac-CoA) and subsequently oxidized in the tricarboxylic acid (TCA) cycle. After synaptic release of glutamate (GLU) by neurons, astrocytes are responsible for most of its uptake via specific high-affinity glutamate transporters to prevent neuronal excitotoxicity, although some pre-synaptic re-uptake can also occur. GLU taken up by astrocytes can be converted to glutamine (GLN) by glutamine synthetase (GS) which can be transferred to neurons where it is transformed into GLU, making it available again for neurotransmission and, in this way, closing the GLU-GLN cycle. The close association between GLU, GLN, and TCA cycle metabolism is indicated in the three cell compartments: GLU can be additionally converted into α-ketoglutarate (α-KG) and be subsequently oxidized. Even though there are reports on the absence of glutamine synthetase and pyruvate carboxylase (PC) in oligodendrocytes, it is not totally clear whether or not they are capable of synthesizing GLN and performing anaplerosis. Finally, neurons are known to synthesize *N*-acetyl-aspartate (NAA) from aspartate (ASP) and Ac-CoA. NAA is thought to be transferred to oligodendrocytes where it is metabolized into ASP and acetate. Whereas the resulting acetate is thought to be extensively used for the synthesis of myelin lipids, the fate of the ASP is still unresolved since it can either be metabolized in oligodendrocytes or transported back to neurons, closing another potentially important metabolic cycle in the brain.

In gray matter, glutamate released from neurons in glutamatergic neurotransmission is mainly taken up by astrocytes (Gegelashvili and Schousboe, 1997, 1998). This drain of glutamate is compensated for by a flow of glutamine from astrocytes to neurons, thus closing the glutamate–glutamine cycle (Figure 1). Net synthesis of tricarboxylic acid (TCA) cycle intermediates and related compounds like glutamate and glutamine depend upon entry of pyruvate *via* an anaplerotic pathway into the TCA cycle. In the brain, this is preferentially or exclusively achieved by pyruvate carboxylase (Patel, 1974a; Waagepetersen et al., 2001), which is not present in neurons but has been shown to be present in astrocytes (Yu et al., 1983; Shank et al., 1985; Cesar and Hamprecht, 1995). This process generates a “new” molecule of oxaloacetate, which may condense with acetyl-CoA to provide net synthesis of the TCA cycle intermediate α-ketoglutarate, from which glutamate can be formed by transamination (Westergaard et al., 1996). Subsequently, glutamine may be synthesized from glutamate (Figure 1) via glutamine synthetase, which like pyruvate carboxylase is exclusively expressed in astrocytes (Martinez-Hernandez et al., 1977; Norenberg and Martinez-Hernandez, 1979). Glutamine released by astrocytes can also function as precursor for the inhibitory neurotransmitter GABA via glutamate (Reubi et al., 1978; Sonnewald et al., 1993). Thus, the concept has been extended to a glutamate–glutamine(–GABA) cycle. The present review aims to further extend these cycles to include oligodendrocytes. There is a clear need for exploring the metabolism of oligodendrocytes in the context of inter-cellular interactions in the brain, involving the major neural cell types, neurons, astrocytes, and oligodendrocytes.

### Tri-cellular compartmentation of brain metabolism

Can the bi-cellular compartmentation of the central nervous system (CNS) described above be extended to a tri-cellular one including oligodendrocytes? The lack of knowledge available on the metabolic role of oligodendrocytes in the brain was highlighted recently with two publications in Nature (Funfschilling et al., 2012; Lee et al., 2012). These studies proposed, for the first time, a link between glycolytic metabolism in oligodendrocytes and axonal integrity and function. Analysis of hexokinase levels in the oligodendroglial fractions isolated from rat brain has shown that in comparison with whole brain or with isolated neurons or astrocytes, oligodendroglia express low levels of this enzyme (Snyder and Wilson, 1983). In agreement with this, Rinholm et al. (2011) suggest that lactate is transported from neurons to oligodendrocytes. Analyzing cell bodies and processes aligned with axons, which were presumed to be oligodendrocytes, a decrease in pH was shown when lactate was applied, thus suggesting that lactate was transported into the cells via MCTs that co-transported H^+^. In a mouse model of globoid cell leukodystrophy (Krabbe disease) an increase in MCT1 was observed in the spinal cord (Meisingset et al., 2013). It should be noted that MCT1 is not only expressed by oligodendrocytes, and that its inhibition in brain has previously been associated with memory deficits due to astrocytic dysfunction (Suzuki et al., 2011). In the white matter, astrocytes are located close to oligodendrocytes and may have different tasks when compared to gray matter astrocytes which are predominantly located near neurons. Disruption of white matter astrocyte–oligodendrocyte–neuronal interaction in multiple sclerosis is described in Cambron et al. (2012).

### Tri-cellular compartmentation of NAA metabolism

Several reviews suggest that metabolism of *N*-acetyl-aspartate (NAA) has a tri-cellular compartmentation (Baslow, 2000; Moffett et al., 2007). Aspartate needed for NAA production can only be synthesized *de novo* in astrocytes (transported to neurons in the form of glutamine), NAA is then assembled and released from neurons and hydrolyzed to acetate and aspartate (Figure 1) by aspartoacylase, which is predominantly located in oligodendroglia (Madhavarao et al., 2004). Leukodystrophies are characterized by degeneration of myelin in the phospholipid layer insulating the axon of a neuron. Canavan’s disease is characterized by an aspartoacylase deficiency and thus accumulation of NAA (Janson et al., 2006). Impairment of oligodendrocyte metabolism leading to accumulation of NAA was also shown in the spinal cord of Twi mice on postnatal day 30 (Meisingset et al., 2013). However, NAA production has also been reported in adult oligodendrocytes, depending on the culturing conditions (Bhakoo and Pearce, 2000). These authors suggest that mature oligodendrocytes in the adult brain synthesize NAA *in vivo*. However, at present there is a lack of consensus regarding oligodendrocyte-mediated NAA synthesis. This controversy needs to be resolved as it has important implications for the interpretation of ^1^HMRS data since NAA levels are commonly used as an indicator of neuronal viability.

### White and gray matter have different energy demands

There is a distinctive difference between white and gray matter astrocytes, moreover, the number of neurons and oligodendrocytes differs considerably between white and gray matter. From these observations it follows that energy demands will vary between these areas. The difference between white and gray matter metabolic intensity has attracted substantial interest. Most authors studying these differences agree that CNS white matter has a lower metabolic intensity than gray matter (Sokoloff, 1977). However, the values published for the cerebral metabolic rate of glucose (CMRglc) calculated from 2-deoxyglucose uptake or cytochrome oxidase activity measurements so far reported differ considerably. Using 2-deoxyglucose uptake in cortex CMRglc is 1–1.6 μmol (g min)^−1^ whereas in white matter it is 0.3–0.4 μmol (g min)^−1^ (references in McKenna et al., 2011). Applying cytochrome oxidase activity measurements to assess brain energy consumption it was proposed that the patterns found in the white matter were similar to those of 2-deoxyglucose uptake in conscious, “resting” animals, although some differences were detected (Hevner et al., 1995). For example, 2-deoxyglucose uptake was found to be approximately threefold higher in gray versus white matter (Sokoloff, 1977) whereas cytochrome oxidase activity showed an 8- to 12-fold difference. These and other discrepancies probably reflect basic technical differences between the two methods used. Compared to 2-deoxyglucose metabolism, the metabolism of cytochrome oxidase is more specific for oxidative metabolism and less so for glycolysis, and more reflective of overall neuronal functional activity occurring over longer time periods lasting hours and weeks, rather than minutes. Measuring oxygen uptake ratios in brain slices, the levels detected were in between those reported above (Hertz and Clausen, 1963). A disparity between glycolysis and oxidative metabolism of glucose in white matter has been found by (Morland et al., 2007). However, the usefulness of ratios is limited since they do not distinguish between axons and non-neuronal cells (oligodendrocytes and astrocytes). Glucose oxidation has been measured in neurons, astrocytes, and oligodendrocytes from the developing brain and these measurements showed that oligodendrocytes oxidized twice as much glucose per hour and mg protein as astrocytes in the TCA cycle and had only slightly less oxidation than neurons (Edmond et al., 1987). In this context it should be mentioned that oligodendrocytes are particularly sensitive to the effects of energy depletion as shown in case reports on patients exposed to CO poisoning (Grunnet and Petajan, 1976; Foncin and Le Beau, 1978; Egan et al., 2004). For example, Foncin and Le Beau (1978) reported an ultrastructural study of biopsy tissue, which showed well preserved and nearly normal cortex morphology, but extensive white matter injury, with disrupted or degenerated myelin and pycnotic oligodendroglia due to CO poisoning. In contrast, the appearance of axons, astrocytes, and capillaries had a nearly unchanged appearance. The destruction affecting oligodendroglia may have been caused by a special vulnerability of oligodendrocytes to glutamate. Similarly in the neonate, glutamate toxicity from hypoxia-ischemia during the perinatal period caused white matter injury and long-term motor and intellectual disability (Fields, 2010).

### The operation of several metabolic pathways remains elusive in oligodendrocytes

Another important question is whether oligodendrocytes also require glutamine from astrocytes. Can oligodendrocytes synthesize glutamine? Glutamine is thought to be an important component of proteins and an excellent energy substrate. Evidence suggests that glutamine synthetase is not present in oligodendrocytes (Pilkington and Lantos, 1982; Derouiche, 2004). Thus, it must be assumed that glutamine from astrocytes reaches the oligodendrocytes, possibly by passing through neurons.

Furthermore, neurons might supply glutamate to the oligodendrocytes or oligodendrocytes might regulate glutamate concentration in extracellular space of the white matter. Glutamate uptake has been shown in oligodendrocytes in the developing brain, but not the adult (DeSilva et al., 2009). Moreover, glutamate receptors are expressed in oligodendrocytes (Karadottir et al., 2005) and also vesicular glutamate release in the white matter has received considerable attention (Karadottir et al., 2005; Kukley et al., 2007; Ziskin et al., 2007).

Do oligodendrocytes express pyruvate carboxylase or are they dependent on aspartate from neurons (via NAA) for the synthesis of oxaloacetate? Aspartate is an essential member of the malate aspartate shuttle which is the major shuttle for reducing equivalents from NADH from glycolysis into the mitochondria (McKenna et al., 2006). It is important to note that aspartate production in neurons is only possible with the help of glutamine from astrocytes (see above). Acetyl-CoA from NAA degradation is used for lipid synthesis in oligodendrocytes and possibly energy production via TCA cycle activity (Figure 1). However, the fate of aspartate from NAA is unclear. It might be (a) metabolized in the TCA cycle in oligodendrocytes, (b) sent back to neurons, or (c) degraded in both cell types (Figure 1). A pivotal question in this context is the localization of pyruvate carboxylase. This enzyme is a nuclear encoded homotetramer found in most eukaryotic and in many prokaryotic tissues and is a member of the family of biotin-dependent carboxylases (Wallace et al., 1998).

It is important to establish whether oligodendrocytes have the possibility to carboxylate pyruvate: if oligodendrocytes express PC they can afford to return aspartate from NAA to the neurons, if they do not, they cannot afford to do so since PC is the only anaplerotic enzyme in the brain (Patel, 1974b). There are a number of publications which propose that PC is exclusively expressed by astrocytes in the CNS (Yu et al., 1983; Shank et al., 1985; Kurz et al., 1993; Cesar and Hamprecht, 1995; McKenna et al., 1995, 2000; Vogel et al., 1998a,b). There is one report that has identified expression of PC in oligodendrocytes (Murin et al., 2009). However, the information in this publication has to be taken with caution since the oligodendrocyte cultures contained 10% astrocytes (Hirrlinger et al., 2002). Evidence for the lack of PC in oligodendrocytes comes from the lack of glycogen phosphorylase in those cells (Richter et al., 1996) since glycogenolysis is a prerequisite for glutamate formation (Gibbs et al., 2007; Sickmann et al., 2012; Xu et al., 2013) probably via dependency of glutamatergic signaling on pyruvate carboxylase (Hertz et al., 2013). Another unresolved question concerning oligodendrocytes is the extent of the pentose phosphate pathway activity. This glucose shunt is active in neurons and astrocytes (Kim et al., 2005; Brekke et al., 2012) and has its highest activity in oligodendrocytes in the developing brain (Edmond et al., 1987) but its activity in mature oligodendrocytes has yet to be investigated. Sykes et al. (1986) suggest that *de novo* synthesis of fatty acids and cholesterol by oligodendrocytes of neonatal rats should be closely geared to the activity of the pentose phosphate pathway in these cells.

## Conclusion

From the limited literature available it can be concluded that vital information about the metabolic capabilities of oligodendrocytes is still missing. With this review we would like to challenge the scientific community to devote time and effort to unraveling what we would like to call oligodendrocyte interaction with astrocytes and neurons (NOA:neuron–oligodendrocyte–astrocyte) interactions. Well-designed experiments are necessary to find answers to these and other questions concerning the referred metabolic pathways and cycles in oligodendrocytes.

## Conflict of Interest Statement

The authors declare that the research was conducted in the absence of any commercial or financial relationships that could be construed as a potential conflict of interest.
